# Engineering LiBH_4_-Based Materials for Advanced Hydrogen Storage: A Critical Review of Catalysis, Nanoconfinement, and Composite Design

**DOI:** 10.3390/molecules29235774

**Published:** 2024-12-06

**Authors:** Yaohui Xu, Yang Zhou, Yuting Li, Maziar Ashuri, Zhao Ding

**Affiliations:** 1 Laboratory for Functional Materials, School of New Energy Materials and Chemistry, Leshan Normal University, Leshan 614000, China; 2 State Key Laboratory of New Textile Materials and Advanced Processing Technology, School of Textile Science and Engineering, Wuhan Textile University, Wuhan 430200, China; 3 Leshan West Silicon Materials Photovoltaic New Energy Industry Technology Research Institute, Leshan 614000, China; 4 College of Materials Science and Engineering, National Engineering Research Center for Magnesium Alloys, National Innovation Center for Industry-Education Integration of Energy Storage Technology, Chongqing University, Chongqing 400044, China; 5 Department of Mechanical, Materials and Aerospace Engineering, Illinois Institute of Technology, Chicago, IL 60616, USA

**Keywords:** lithium borohydride, hydrogen storage, catalysis, nanoconfinement, reactive composites, thermodynamic destabilization

## Abstract

Lithium borohydride (LiBH_4_) has emerged as a promising hydrogen storage material due to its exceptional theoretical hydrogen capacity (18.5 wt.%). However, its practical application is hindered by high dehydrogenation temperature (>400 °C), sluggish kinetics, and limited reversibility due to stable intermediate formation. This review critically analyzes recent advances in LiBH_4_ modification through three primary strategies: catalytic enhancement, nanostructure engineering, and reactive composite design. Advanced carbon architectures and metal oxide catalysts demonstrate significant improvements in reaction kinetics and cycling stability through interface engineering and electronic modification. Sophisticated nanostructuring approaches, including mechanochemical processing and infiltration techniques, enable precise control over material architecture and phase distribution, effectively modifying thermodynamic and kinetic properties. The development of reactive hydride composites, particularly LiBH_4_-MgH_2_ systems, provides promising pathways for thermodynamic destabilization while maintaining high capacity. Despite these advances, challenges persist in maintaining engineered structures and suppressing intermediate phases during cycling. Future developments require integrated approaches combining multiple modification strategies while addressing practical implementation requirements.

## 1. Introduction

The global imperative to transition toward sustainable energy systems has positioned hydrogen as a pivotal energy carrier for the 21st century. With its exceptional gravimetric energy density (142 MJ/kg), clean combustion characteristics, and versatility across various applications, hydrogen presents a promising pathway for decarbonizing multiple sectors, including transportation, industrial processes, and power generation [1,2,3,4,5]. However, the widespread adoption of hydrogen-based technologies faces a fundamental challenge: the development of efficient, safe, and economically viable hydrogen storage systems [6,7,8,9]. To guide research and development efforts in this critical area, the U.S. Department of Energy has established specific technical targets for onboard hydrogen storage systems, as shown in Table 1. These ambitious targets underscore the significant technical challenges that must be overcome to enable practical hydrogen storage solutions. Conventional storage methods—compressed gas and cryogenic liquefaction—present significant limitations for practical applications [10,11,12]. Compressed hydrogen storage requires extremely high pressures (350–700 bar), necessitating expensive composite tanks and raising safety concerns, while cryogenic storage, despite achieving higher volumetric densities, demands considerable energy for liquefaction (−253 °C) and suffers from continuous boil-off losses. These challenges have catalyzed intensive research into solid-state hydrogen storage materials as alternative solutions [13,14,15,16,17].

Among the diverse array of solid-state hydrogen storage candidates, complex hydrides have emerged as particularly promising materials due to their exceptional gravimetric and volumetric hydrogen densities. Within this class, lithium borohydride (LiBH_4_) stands out as a remarkable candidate, offering one of the highest theoretical hydrogen storage capacities (18.5 wt.%) among known materials [18,19]. Table 2 summarizes the key advantages and challenges associated with LiBH_4_ as a hydrogen storage material. Despite its promising characteristics, the practical implementation of LiBH_4_-based storage systems faces several critical challenges [20,21,22]. The material exhibits high thermodynamic stability, requiring temperatures exceeding 400 °C for hydrogen desorption—far beyond the operational range of most applications, particularly proton exchange membrane fuel cells (PEMFCs) that operate optimally below 100 °C. Furthermore, the dehydrogenation process is complicated by the formation of stable intermediate species, notably Li_2_B_12_H_12_, which impedes complete hydrogen release and system reversibility. The rehydrogenation process demands severe conditions (typically > 600 °C and >150 bar H_2_), presenting additional engineering challenges for practical applications.

Recent years have witnessed significant advances in addressing these limitations through various materials engineering strategies, which can be broadly categorized into three main directions: catalyst doping to reduce kinetic barriers and modify reaction pathways; nanostructuring and confinement to enhance surface reactivity and alter thermodynamic properties; and the formation of reactive hydride composites to modify the overall reaction energetics [23,24,25,26]. Each strategy has demonstrated promising results while also revealing new challenges and opportunities for further optimization. These advances have been supported by the development of advanced characterization techniques and mechanistic studies, enabling a deeper understanding of the fundamental processes governing hydrogen storage in LiBH_4_-based systems.

While several comprehensive reviews have previously examined LiBH4-based systems, the rapid evolution of this field necessitates an updated perspective that integrates recent breakthroughs. This review uniquely synthesizes the latest advances (2019–2024) in catalyst design, nanostructuring, and composite development, with a particular emphasis on the fundamental mechanisms governing performance enhancement. By focusing on the critical role of interface engineering and phase evolution during cycling, along with the synergistic effects of combined modification strategies, we provide deeper insights into practical system development than previously available in the literature. This comprehensive review provides a critical analysis of the current state of LiBH_4_-based hydrogen storage systems, examining both fundamental aspects and practical considerations. Following a detailed exploration of the structural and thermodynamic properties of LiBH_4_, we systematically evaluate various modification strategies, including mechanistic studies of catalyst effects, nanoconfinement approaches, and reactive hydride composite systems. Special attention is devoted to emerging trends and innovative approaches in LiBH_4_ modification, including the development of multifunctional catalysts, advanced nanostructuring techniques, and novel composite systems. Throughout this review, we emphasize the interplay between material properties, processing conditions, and system performance, providing insights for future research directions.

This review concludes with a comprehensive assessment of the remaining challenges and future opportunities in LiBH_4_-based hydrogen storage research. By analyzing the critical developments needed to bridge the gap between current capabilities and practical requirements for commercial applications, this review serves as a valuable resource for researchers, engineers, and decision-makers working toward the realization of efficient hydrogen storage systems based on LiBH_4_ and related materials. The systematic examination of recent advances, coupled with a critical analysis of persistent challenges, provides a foundation for future research efforts aimed at developing practically viable LiBH_4_-based hydrogen storage systems.

## 2. Structural Chemistry and Reaction Mechanisms of LiBH_4_

**Figure 1:**
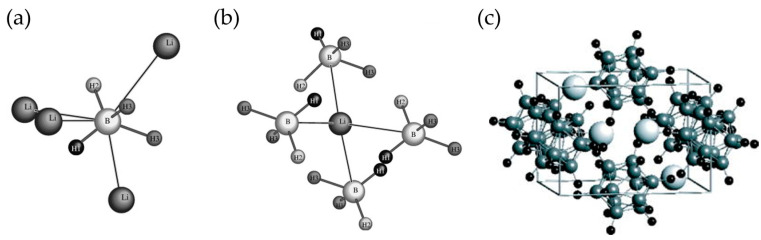
The LiBH4 structure at room temperature (**a**) Boron (top) and (**b**) lithium (bottom) [27]. (**c**) Atomistic structure model of the monoclinic Li_2_B_12_H_12_ space group: P21/n, Z = 2; a = 7.358 Å, b = 9.556 Å, c = 6.768 Å, and β = 92.26°. The large, middle, and small spheres denote Li, B, and H atoms, respectively [38].

The fundamental behavior of LiBH_4_ as a hydrogen storage material is governed by its unique structural chemistry and complex reaction mechanisms. At room temperature, LiBH_4_ crystallizes in an orthorhombic structure (Figure 1a,b), characterized by a highly ordered network where each [BH_4_]^−^ anion is tetrahedrally coordinated by four lithium cations [27]. This distinctive arrangement creates a dual bonding environment: strong covalent B-H bonds within the [BH_4_]^−^ tetrahedra and ionic interactions between Li^+^ and [BH_4_]^−^ units [28,29]. The interplay between these bonding types not only contributes to the material’s exceptional hydrogen storage capacity of 18.5 wt.% but also significantly influences its thermal stability and hydrogen release characteristics [30,31,32].

The structural evolution of LiBH_4_ during heating reveals complex phase behavior that directly impacts its hydrogen storage properties. Upon heating, LiBH_4_ undergoes a first-order phase transition from its low-temperature orthorhombic phase to a high-temperature hexagonal phase at approximately 380 °C [33,34]. This phase transformation is accompanied by dramatic changes in the material’s physical properties, including a significant increase in ionic conductivity and enhanced hydrogen mobility within the crystal lattice. The heightened atomic and ionic mobility in the high-temperature phase facilitate the initial stages of hydrogen release, though the temperature requirement remains substantially above the practical operating range for most applications.

The dehydrogenation of LiBH_4_ proceeds through a complex, multi-step pathway that involves the formation of several intermediate phases [33,35,36,37]. The process begins at temperatures above 400 °C, following the sequence below.

- Step 1: LiBH_4_ → 1/12 Li_2_B_12_H_12_ + 5/6 LiH + 13/12 H_2_ (T > 400 °C)
- Step 2: 1/12 Li_2_B_12_H_12_ → 1/10 Li_2_B_10_H_10_ + 1/15 LiH + 1/30 H_2_ (T > 450 °C)
- Step 3: 1/10 Li_2_B_10_H_10_ + 9/10 LiH → LiBH + 9/20 H_2_ (T > 500 °C)
- Step 4: LiBH → LiH + B + 1/2 H_2_ (T > 550 °C)

Among these steps, the formation of Li_2_B_12_H_12_ (Figure 1c) represents a critical turning point in the dehydrogenation process [38]. This intermediate compound exhibits remarkable stability due to its unique icosahedral structure (space group: P21/n, Z = 2; a = 7.358 Å, b = 9.556 Å, c = 6.768 Å, β = 92.26°) and extensive electron delocalization within its closed-cage architecture. The extraordinary stability of Li_2_B_12_H_12_ stems from its highly symmetric structure, where 12 (BH) units form a nearly perfect icosahedron, creating a network of strong B-B bonds that resist further decomposition. This structural characteristic presents a significant kinetic barrier to both complete dehydrogenation and subsequent rehydrogenation processes.

The thermodynamic landscape of LiBH_4_ decomposition is equally complex. The standard enthalpy of dehydrogenation (ΔH ≈ 74 kJ/mol H_2_) reflects the considerable energy requirement for breaking B-H bonds and rearranging the crystal structure [37,39,40,41]. This high enthalpic barrier, combined with the formation of stable intermediates, necessitates temperatures well above 400 °C for appreciable hydrogen release [42,43]. The situation becomes even more challenging during rehydrogenation, which typically requires hydrogen pressures exceeding 100 bar and temperatures above 400 °C. The rehydrogenation pathway can be represented as follows:

LiH + B + 3/2 H_2_ → LiBH + 1/2 H_2_ → 1/10 Li_2_B_10_H_10_ + 9/10 LiH → 1/12 Li_2_B_12_H_12_ + 5/6 LiH + 1/12 H_2_ → LiBH_4_

The severity of these rehydrogenation conditions stems from multiple factors, including the thermodynamic stability of the dehydrogenated products (LiH and B), the kinetic barriers associated with B-H bond formation, and the necessity of decomposing stable intermediate phases. The reformation of the original LiBH_4_ structure requires precise control over reaction conditions to promote the complete reversal of the dehydrogenation pathway while minimizing the formation of persistent intermediate phases.

The structural evolution during cycling between hydrogenated and dehydrogenated states introduces additional complexity. Repeated hydrogen cycling can lead to structural deterioration, phase segregation, and the accumulation of stable intermediates, particularly Li_2_B_12_H_12_ and Li_2_B_10_H_10_. These changes often result in capacity degradation and slower kinetics in subsequent cycles. Understanding these structural and mechanistic aspects is crucial for developing effective strategies to enhance the hydrogen storage performance of LiBH_4_. The challenges posed by the formation of stable intermediates and the high thermodynamic barriers have motivated extensive research into various modification approaches, including catalyst incorporation, nanostructuring, and the development of reactive hydride composites, which will be examined in detail in subsequent sections.

## 3. Catalytic Enhancement Strategies

Catalyst doping is a widely researched approach aimed at enhancing the dehydrogenation and rehydrogenation kinetics of LiBH_4_ as well as lowering its dehydrogenation temperature. Studies have shown that introducing catalysts into LiBH4 can facilitate the breaking and reformation of B-H and Li-H bonds, thereby reducing activation energy and improving the kinetics of hydrogen absorption and desorption. Various catalysts, including carbon materials and metal oxides, have been explored for their effects on the hydrogen storage properties of LiBH_4_ [44]. The catalytic effects are attributed to several key mechanisms. First, catalysts can act as nucleation sites for the formation of intermediate compounds, such as Li_2_B_12_H_12_ and Li_2_B_10_H_10_, reducing the activation energy required for the formation and decomposition of these compounds. Second, catalysts interact with B-H and Li-H bonds, weakening their bond strength and promoting bond dissociation during the dehydrogenation process. Finally, catalysts can facilitate the recombination of dehydrogenated products, such as LiH and B, during the rehydrogenation process, thus enhancing the reversibility of the hydrogen storage system. Although significant improvements in the hydrogen storage properties of LiBH_4_ have been achieved through catalyst doping, challenges remain, such as the long-term stability and recyclability of catalysts during hydrogen absorption and desorption cycles [45]. Additionally, catalysts may sometimes promote the formation of stable intermediate compounds (e.g., Li_2_B_12_H_12_), which hinder the complete dehydrogenation and rehydrogenation of LiBH_4_ [46]. Therefore, the selection and optimization of catalysts for LiBH_4_-based hydrogen storage systems require an in-depth understanding of catalytic mechanisms and the structure–property relationships involved. Table 3 summarizes the hydrogen storage performance of various composite materials under different temperatures and conditions, showing that the desorption capacity varies with the type of additive and experimental parameters. Overall, higher temperatures and vacuum conditions generally result in increased desorption capacities, with different additives demonstrating significant effects on performance modulation. These findings offer valuable insights for the optimization of hydrogen storage materials.

**Table 3:** Hydrogen storage and desorption capacities of LiBH_4_-based composite materials under various conditions.

Materials	Adsorb Temperature	Adsorb Condition	Adsorb Capacities (ad)	Desorb Temperature	Desorb Condition	Desorb Capacities	Refs.
LiBH_4_-30 wt.% SWNTs	-	-	-	550 °C	Vacuum	12.3 wt.%	[47]
LiBH_4_-30 wt.% G	\	\	\	450 °C	Vacuum	6.8 wt.%	[48]
LiBH_4_-30 wt.% AC	\	\	\	450 °C	Vacuum	10 wt.%	
LiBH_4_-30 wt.% SWNTs	\	\	\	450 °C	Vacuum	11 wt.%	
LiBH_4_-D-carbon	\	\	\	380 °C	Vacuum	7.4 wt.%	[49]
LiBH_4_-AC-carbon	\	\	\	380 °C	Vacuum	7.6 wt.%	[50]
LiBH_4_-20 wt.% Grapene	\	\	\	700 °C	Vacuum	11.4 wt.%	[51]
LiBH_4_@NiO_Ar	150 °C	10 bar	2.13 wt.%	450 °C	Vacuum	6.32 wt.%	[52]
LiBH_4_-0.06TiO	500 °C	50 bar/100 min	9 wt.%	400 °C	Vacuum/10 min	9 wt.%	[53]
LiBH_4_-Fe_3_O_4_	450 °C	10 MPa/60 min	6.7 wt.%	400 °C	Vacuum	8.1 wt.%	[54]
LiBH_4_-30% Fe_3_O_4_	450 °C	10 MPa/60 min	8.1 wt.%	350 °C	Vacuum/30 min	7.8 wt.%	[55]
LiBH_4_-SrF_2_	450 °C	8 MPa	5.2 wt.%	550 °C	Vacuum	5.3 wt.%	[56]
LiBH_4_-20 wt.% Ce_2_S_3_	\	\	\	400 °C	Vacuum/50 min	4 wt.%	[57]

### 3.1. Carbon-Based Architectures

Carbon-based materials have emerged as exceptional catalytic platforms for LiBH_4_ modification, offering unique advantages through their tunable structure, high surface area, and versatile surface chemistry. Beyond their traditional role as supports, carbon materials demonstrate intrinsic catalytic activity through electronic interactions and the creation of specialized reactive sites for hydrogen storage processes.

The pioneering work by Fang et al. [47] revealed the remarkable potential of single-walled carbon nanotubes (SWNTs) in enhancing LiBH_4_ performance. Through systematic ball milling studies, they demonstrated that SWNTs significantly improve dehydrogenation/re-hydrogenation kinetics, enabling reversible hydrogen storage under milder conditions. Subsequent comparative analyses of various carbon architectures, including graphite (G), purified SWNTs, and activated carbon (AC), established a clear structure–performance relationship [48]. Both SWNTs and AC exhibited superior catalytic effects compared to graphite, highlighting the importance of surface accessibility and nanoscale architecture in catalytic efficiency. Further advancement in carbon-based catalysis was achieved through the strategic incorporation of multi-walled carbon nanotubes (MWCNTs). Weng et al. demonstrated that optimized MWCNT content (>7 wt.%) prevents agglomeration and facilitates approximately 7.5 wt.% hydrogen release. The enhanced performance was attributed to the creation of efficient gas diffusion channels and increased contact area between LiBH_4_ and H_2_, illustrating the critical role of architectural design in catalyst optimization [49].

**Figure 2:**
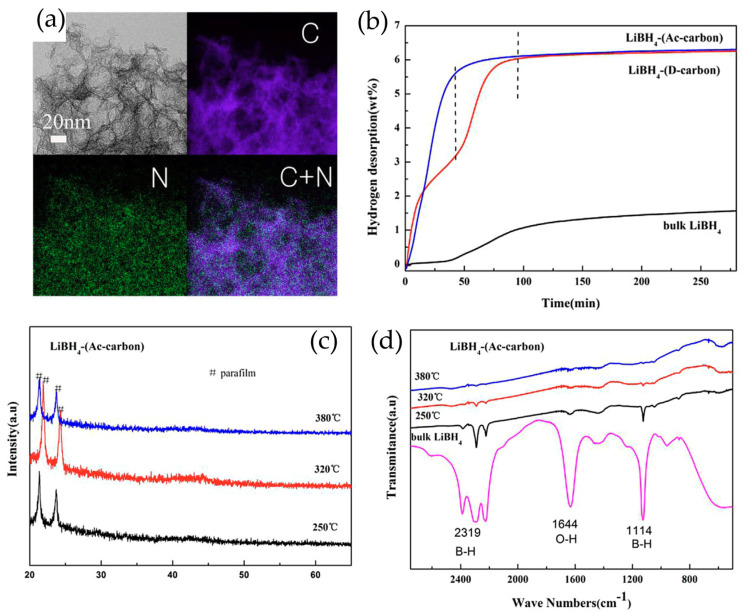
(**a**) TEM image and the corresponding C- and N-elemental mappings of D-carbon. (**b**) Isothermal hydrogen desorption curves at 320 °C of LiBH_4_-(D-carbon), LiBH4-(Ac-carbon), and bulk LiBH_4_. (**c**) XRD patterns of dehydrogenated LiBH4-(Ac-carbon) at 250 °C, 320 °C, and 380 °C for 5 h. (**d**) FT-IR spectra of the dehydrogenated products of LiBH_4_-(Ac-carbon) at 250 °C, 320 °C and 380 °C for 5 h; pure LiBH_4_ is also included for comparison [50].

A significant breakthrough in carbon catalyst design was achieved by Zhao et al. [50] through the development of nitrogen-doped hierarchical porous carbon (NHPC) materials using a zeolitic imidazolate framework (ZIF-8) as a sacrificial template. This innovative approach yielded a three-dimensional carbon framework with exceptional characteristics: high nitrogen content (11.22%), large surface area (2477 m^2^·g⁻^1^), and hierarchical porosity. As shown in Figure 2a, the overlap of N signals with the main C signals confirms the presence of nitrogen doping. Ball-milled composites of LiBH4 with these carbon materials demonstrated remarkable hydrogen release properties, achieving approximately 6.13 wt.% H_2_ release within 100 min at 320 °C (Figure 2b). The detailed structural characterization through XRD and FT-IR spectroscopy (Figure 2c,d) revealed that the carbon framework not only catalyzes hydrogen release but also stabilizes the dehydrogenated state while maintaining system reversibility.

The integration of graphene into LiBH_4_ systems has opened new avenues for performance enhancement. Xu et al. [51] achieved a significant reduction in initial hydrogen release temperature to 230 °C by incorporating 20 wt.% graphene, resulting in a total weight loss of 11.4 wt.% below 700 °C. The graphene modification led to a remarkable decrease in dehydrogenation enthalpy from 74 kJ mol⁻^1^ H₂ to 40 kJ mol⁻^1^ H_2_, demonstrating the profound impact of two-dimensional carbon architectures on the thermodynamics of hydrogen storage. Under hydrogen pressure of 3 MPa, the composite maintained a capacity of approximately 4.0 wt.% over 10 cycles at 400 °C, though the formation of Li_2_B_10_H_10_ during rehydrogenation highlighted ongoing challenges in achieving complete reversibility.

The exceptional performance enhancements achieved through carbon-based catalytic systems stem from a sophisticated interplay of multiple mechanisms. The hierarchical architecture of carbon materials creates an intricate network of active sites and diffusion pathways, while their tunable surface chemistry enables precise control over hydrogen dissociation and recombination processes. Surface functionalization, particularly nitrogen doping, introduces additional electronic modification effects that can significantly alter local reaction energetics. Furthermore, the thermal conductivity of carbon frameworks facilitates efficient heat distribution during cycling, promoting uniform reaction progression. However, maintaining architectural integrity during extended cycling remains challenging, particularly regarding the stability of engineered interfaces and pore structures. The complex balance between maximizing catalytic activity through increased surface area and maintaining structural stability during repeated hydrogen cycling necessitates the careful optimization of carbon architecture design. Understanding and controlling these structure–property relationships is crucial for developing next-generation carbon-based catalytic systems that can maintain their enhancement effects throughout the material’s operational lifetime.

### 3.2. Metal Oxide Catalysis

Metal oxide catalysts have emerged as powerful modifiers for LiBH_4_ systems, offering unique advantages through their diverse crystal structures, tunable surface chemistry, and versatile redox properties [17,58]. The incorporation of metal oxides can significantly alter the thermodynamics and kinetics of hydrogen storage processes through multiple mechanisms, including surface activation, electronic modification, and the creation of reactive interfaces [58,59]. The rational design of oxide catalysts, particularly through morphological control and interface engineering, has led to remarkable improvements in LiBH_4_ performance.

The development of nickel oxide-based catalysts exemplifies the importance of morphological control in oxide catalyst design. Kaliyaperumal et al. [52] demonstrated the exceptional potential of flower-like NiO structures synthesized through a controlled hydrothermal process. When combined with LiBH_4_ via wet sonication, these architecturally designed catalysts enabled hydrogen release initiation at temperatures as low as 250 °C. The composite system achieved an impressive two-stage hydrogen release: 2.13 wt.% H_2_ between 50–250 °C, followed by an additional 6.32 wt.% H_2_ in the range of 275–450 °C. This stratified release pattern, significantly superior to unmodified LiBH_4_’s performance, highlights the profound impact of catalyst morphology on hydrogen storage behavior.

**Figure 3:**
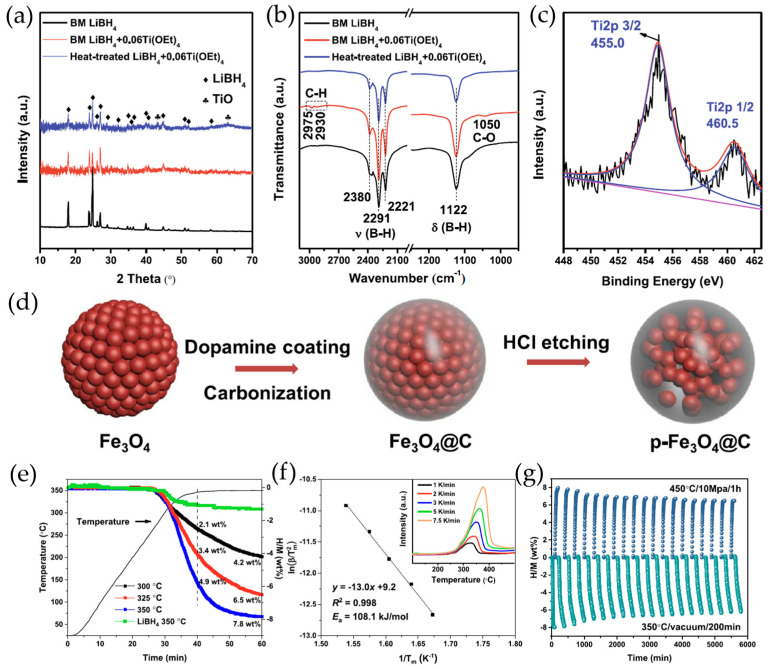
(**a**) XRD patterns and (**b**) FTIR spectra of the heat-treated LiBH4 + 0.06Ti(OEt)_4_ mixture at 230 °C for 30 min as well as ball-milled mixture and ball-milled LiBH_4_. (**c**) XPS spectrum of Ti 2p of the heat-treated LiBH_4_ + 0.06Ti(OEt)_4_ mixture. (**d**) Schematic illustration of the preparation process of the passionfruit-like p-Fe_3_O_4_@C hybrid. (**e**) Isothermal dehydrogenation curves of the 6LiBH_4_@4p-Fe_3_O_4_@C system at 300 °C, 325 °C, and 350 °C and that of the pristine LiBH_4_ at 350 °C. (**f**) Kissinger’s plot of the 6LiBH_4_@4p-Fe_3_O_4_@C system, where the inset is the TPD-MS curves measured at different heating rates. (**g**) Cyclic curves of the 6LiBH4@4p-Fe_3_O_4_@C system with a regime of dehydrogenation at 350 °C for 200 min and hydrogenation at 450 °C under 10 MPa H_2_ for 100 min [55].

Titanium-based oxide systems have demonstrated particularly promising results through precise compositional and structural control. Li et al. [53] developed an innovative LiBH_4_-0.06TiO composite system that showcases the complex interplay between catalyst structure and performance. An X-ray diffraction analysis post-ball milling revealed the preservation of the LiBH_4_ phase, while FTIR spectroscopy (Figure 3a,b) identified characteristic absorption peaks at 2975, 2930, and 1050 cm⁻^1^ corresponding to the -CH_3_, -CH_2_, and C-O groups from the titanium precursor. Upon thermal treatment, the emergence of TiO peaks coincided with the disappearance of organic functional groups, indicating successful structural transformation. The modified system demonstrated remarkable performance improvements, with hydrogen desorption initiating at 240 °C and reaching its peak at 340 °C—a substantial 140 °C reduction compared to pristine LiBH_4_. Under isothermal conditions at 400 °C, the composite rapidly released 9 wt.% H_2_ within 10 min. The system’s rehydrogenation capability was equally impressive, initiating hydrogen uptake at 150 °C and achieving a 9 wt.% capacity after 100 min at 500 °C under 50 bar H_2_. An XPS analysis (Figure 3c) confirmed the presence of TiO through characteristic spin-orbit splitting peaks at 455.0 and 460.5 eV, providing valuable insights into the catalyst’s electronic state.

Iron oxide-based catalysts have revealed fascinating mechanistic insights through both theoretical and experimental investigations. Liu et al. [60] employed first-principles calculations to elucidate the fundamental role of Fe_2_O_3_ clusters in modifying the LiBH_4_ (001) surface. Their computational studies unveiled the critical coupling effect between spin-up Fe-d orbitals and the hybridization of H-s, B-p, and Fe-d orbitals, establishing Fe_2_O_3_ clusters as effective nucleation sites for surface activation. These theoretical insights have guided the development of advanced iron oxide architectures. Wang et al. [54] successfully synthesized myrica-like hierarchical iron oxide-graphene nanospheres that enabled hydrogen release below 100 °C and achieved an impressive total capacity of 8.1 wt.% H_2_ at 400 °C, maintaining 70% capacity after 10 cycles.

Further advancement in iron oxide catalyst design was achieved through the development of passionfruit-like porous hybrid materials (p-Fe_3_O_4_@C) by Wang et al. [55]. This innovative architecture, featuring ultrafine Fe_3_O_4_ particles encapsulated within carbon shells, demonstrated the power of synergistic effects combining nano-confinement, catalysis, and surface instability. With optimized LiBH_4_ loading at 60 wt.%, the system achieved a remarkably low dehydrogenation onset temperature of 175 °C and released 4.9 wt.% H_2_ by 350 °C. The composite demonstrated exceptional kinetics, reaching 7.8 wt.% capacity within 20 min and maintaining stability through extended isothermal operation. A detailed kinetic analysis revealed a significant reduction in dehydrogenation activation energy to 108.1 kJ mol⁻^1^, substantially lower than the 179.1 kJ mol⁻^1^ of unmodified LiBH_4_. The system’s cycling stability was particularly noteworthy, retaining 93% capacity after 20 cycles at 300 °C (Figure 3e–g).

The remarkable effectiveness of metal oxide catalysts in enhancing LiBH4’s hydrogen storage performance arises from their multifaceted influence on both reaction thermodynamics and kinetics. The formation of reactive metal–oxygen interfaces creates localized electronic environments that facilitate hydrogen dissociation and recombination, while the potential for oxygen ion mobility enables the dynamic modification of local bonding environments. The observed synergistic effects between different oxide phases, particularly in hierarchical architectures combining multiple metal species, demonstrate the potential for further performance optimization through careful catalyst design. The formation of oxygen-containing intermediates during cycling can modify reaction pathways and influence the stability of various phases, though controlling these processes to maintain consistent performance remains challenging. The development of oxide catalysts that can simultaneously address multiple performance limitations while maintaining structural and chemical stability under cycling conditions represents a critical direction for future research in this field.

### 3.3. Alternative Catalyst Systems

Beyond traditional carbon and oxide catalysts, a diverse array of alternative materials including borides, fluorides, and sulfides has demonstrated remarkable potential in modifying LiBH_4_’s hydrogen storage properties [61,62]. These compounds offer unique advantages through their distinctive chemical bonding, electronic structures, and surface properties, enabling novel pathways for performance enhancement. The systematic investigation of these alternative catalyst systems has revealed innovative strategies for overcoming the limitations of conventional approaches while providing deeper insights into catalyst design principles [63].

**Figure 4:**
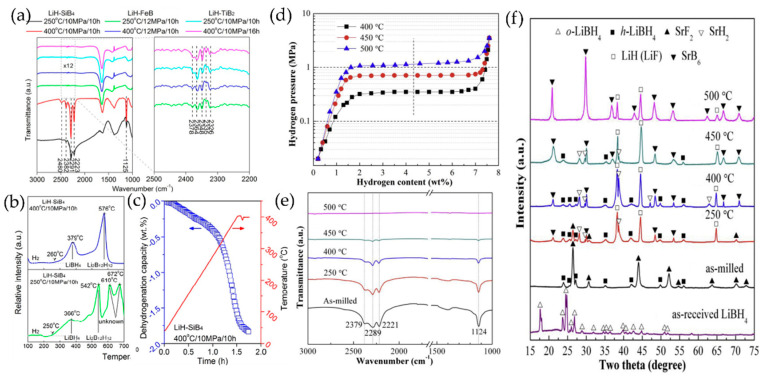
(**a**) FTIR spectra of SiB_4_/FeB/TiB_2_LiH after hydrogenation at different conditions. The right part is the enlargement of the dashed region in left FTIR, TPD-MS, (**b**) and isothermal dehydrogenation curve at 400 °C (**c**) of SiB_4_-LiH samples after hydrogenation at different conditions [64]. (**d**) Dehydrogenation of PeC isotherms of the 6LiBH_4_/SrF_2_ system at 400, 450, and 500 °C. (**e**) FTIR spectra of the 6LiBH_4_/SrF_2_ system. (**f**) XRD patterns of different samples [56].

Boride-based catalysts have emerged as particularly effective modifiers due to their chemical compatibility and unique electronic properties. Cai et al. [64] conducted a comprehensive investigation of SiB_4_/FeB/TiB_2_ additives, revealing their profound impact on LiBH_4_’s reversible hydrogenation behavior. An infrared spectroscopic analysis (Figure 4) demonstrated the formation of characteristic B-H bonds in the 2200–2500 cm⁻^1^ region for SiB_4_/FeB/TiB_2_-LiH systems. The SiB_4_-LiH combination proved especially effective, exhibiting distinctive B-H stretching vibrations at 2382, 2291, and 2223 cm⁻^1^, along with a characteristic bending mode at 1125 cm⁻^1^. These spectral features, displaying notably higher intensities than conventional systems, indicated enhanced LiBH4 formation kinetics. While the presence of a B-H vibration at 2480 cm⁻^1^ signaled the formation of the intermediate Li_12_B_12_H_12_ phase, its relative concentration remained manageable. The composite demonstrated complex thermal behavior, with both LiBH_4_ and Li_12_B_12_H_12_ phases exhibiting dehydrogenation at reduced temperatures, as shown in Figure 4b. However, the system’s overall hydrogen release capacity at 400 °C was limited to 1.8 wt.%, corresponding to approximately 19.6 wt.% LiBH_4_ formation during hydrogenation (Figure 4c).

Fluoride-based systems have introduced novel approaches to the thermodynamic modification of LiBH_4_. Zhao et al. [56] investigated the 6LiBH_4_/SrF_2_ system and revealed distinctive plateau regions in the pressure–composition isotherms at approximately 0.35, 0.70, and 1.15 MPa for temperatures of 400, 450, and 500 °C, respectively (Figure 4d). A detailed thermodynamic analysis determined enthalpy and entropy changes for dehydrogenation at 52 kJ/mol H_2_ and 87 J/(mol·K) H_2_, respectively, representing a significant reduction in LiBH_4_’s thermal stability. The system demonstrated practical reversibility, achieving LiBH_4_ and SrF_2_ regeneration alongside LiSrH3 formation at 450 °C under 8 MPa hydrogen pressure, with approximately 5.2 wt.% hydrogen release capacity maintained in the second dehydrogenation cycle. Mechanistic studies using XRD and FTIR techniques revealed complex phase evolution during ball milling and thermal treatment. The process was initiated with anion substitution between reactants at ambient temperature, followed by the sequential formation of SrH_2_, LiH (LiF), and SrB_6_ phases upon heating. The reaction pathway culminated in the conversion to SrF_2_ and LiH (LiF) at 500 °C, proceeding through the following reaction: 6LiBH_4_ + SrF_2_ → SrB_6_ + 2LiF + 4LiH + 10H_2_ (Figure 4e).

Sulfide catalysts have demonstrated unique capabilities in enhancing LiBH_4_’s performance through distinct mechanistic pathways. Wang et al. [57] developed Ce_2_S_3_ particles with controlled dimensions of 100–200 nm using a sophisticated solvothermal approach. The interaction between Ce_2_S_3_ and LiBH_4_ generated Li_2_S and CeB_6_ as dehydrogenation products, which functioned as active species during rehydrogenation, significantly enhancing the system’s reversibility. At an optimal Ce_2_S_3_ loading of 20 wt.%, the composite maintained a hydrogen absorption capacity of 3.6 wt.% even after four cycles. The modification reduced the initial dehydrogenation temperature to 250 °C, representing an 80 °C improvement over pristine LiBH_4_. Under isothermal conditions at 400 °C, the composite released approximately 4.0 wt.% hydrogen within 3000 s, representing a 67% improvement over the unmodified system. The activation energy for dehydrogenation decreased from 181.80 kJ/mol to 157.82 kJ/mol, confirming the catalytic effectiveness of the sulfide system.

The investigation of alternative catalyst systems, encompassing borides, fluorides, and sulfides, has revealed sophisticated mechanisms for performance enhancement that complement traditional approaches. These materials demonstrate unique capabilities in modifying both the thermodynamic landscape and reaction pathways of LiBH_4_ through distinct chemical interactions and interface effects. The formation of intermediate phases during cycling can create self-sustaining catalytic effects, while careful control over composition and structure enables the targeted modification of specific reaction steps. However, the complexity of these systems, particularly regarding phase evolution and interface stability during extended cycling, presents ongoing challenges for practical implementation. The continued development of these alternative catalysts requires careful consideration of the balance between catalytic effectiveness, system complexity, and long-term stability. Understanding the fundamental mechanisms of catalyst–LiBH_4_ interactions at the atomic and molecular levels will be crucial for optimizing these systems for practical hydrogen storage applications.

## 4. Nanostructure Engineering

The development of nanostructured LiBH_4_ represents a transformative approach to addressing the fundamental limitations of bulk material systems. Through precise control of material architecture at the nanoscale, significant modifications to thermodynamic stability, reaction kinetics, and mass transport characteristics can be achieved. Nanostructuring strategies operate through multiple synergistic mechanisms: increased surface-to-volume ratio facilitating enhanced hydrogen molecule interactions; shortened diffusion pathways for hydrogen atoms and ions; modified local chemical environments affecting bond strengths and reaction energetics; and controlled interface engineering for improved reaction kinetics. The successful implementation of nanostructuring approaches requires careful consideration of synthesis methods, stability under cycling conditions, and the preservation of effective hydrogen storage capacity. Table 4 summarizes the hydrogen storage performance of various composite materials prepared using different synthesis methods during adsorption and desorption processes. The results indicate that the choice of preparation method and experimental conditions significantly influences material performance. Notably, there is a marked variation in capacities under high-pressure adsorption and vacuum desorption conditions, with certain materials demonstrating superior adsorption or desorption efficiency. These findings provide a foundational reference for the optimization of hydrogen storage materials.

**Table 4:** Hydrogen storage performance of LiBH_4_-based composites prepared by different methods under various conditions.

Materials	Method	Absorb Temperature	Absorb Condition	Absorb Capacities	Desorb Temperature	Desorb Condition	Desorb Capacities	Refs.
LiBH_4_-30 wt.% FL-Grs	Ball Milling	350 °C	100 bar/60 min	7 wt.%	350 °C	Vacuum/100 min	7.8 wt.%	[65]
LiBH_4_@CNCs	Melt	400 °C	50 bar/30 min	4 wt.%	400 °C	Vacuum/60 min	4.8 wt.%	[66]
LiBH_4_@NPC	Melt	\	\	\	350 °C	Vacuum/120 min	6.5 wt.%	[67]
LiBH_4_/AC	Melt	350 °C	6 MPa	6 wt.%	400 °C	Vacuum	13.6 wt.%	[68]
50LBH4@NC-NbF_5_	Melt	300 °C	12 MPa/100 min	6.2 wt.%	320 °C	Vacuum/39 min	7.55 wt.%	[69]
LiBH_4_/carbon	Solution	\	\	\	300 °C	Vacuum/90 min	3.4 wt.%	[70]
LiBH_4_@2TiO_2_	Solution	\	\	\	310 °C	Vacuum/120 min	2.5 wt.%	[71]
LiBH_4_@NiMnO_3_	Solution	\	\	\	300 °C	Vacuum/60 min	2.8 wt.%	[72]
Nano-LiBH_4_	Solution	400 °C	100 bar/25 min	12 wt.%	450 °C	Vacuum/100 min	13 wt.%	[73]

### 4.1. Mechanochemical Processing

High-energy ball milling has emerged as a sophisticated mechanochemical technique for engineering nanostructured LiBH_4_ systems [74]. Beyond simple particle size reduction, this approach enables precise control over defect chemistry, interface formation, and local structural modifications. Through careful optimization of processing parameters, ball milling can induce fundamental changes in material properties while maintaining high hydrogen storage capacity.

**Figure 5:**
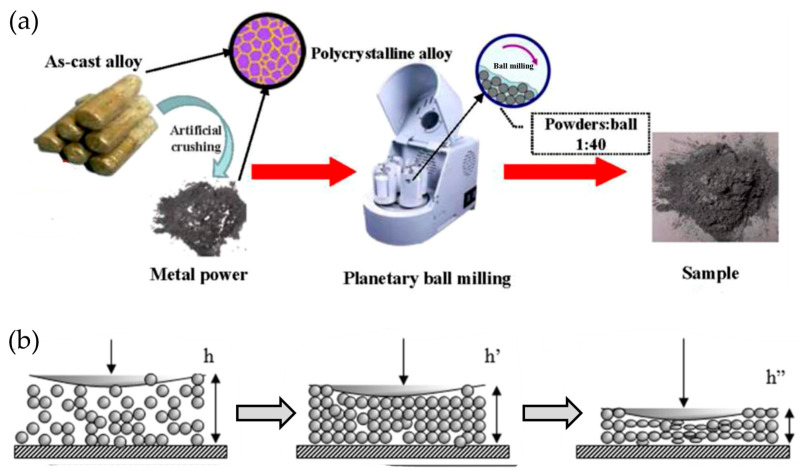
(**a**) The flowchart for the preparation method of the as-milled Sm_5_Mg_41_ alloy [75]. (**b**) Illustration of the deformation of powder agglomerate during the impact process [76].

The mechanistic complexity of ball milling processes is illustrated through the multi-dimensional forces applied during processing. As shown in Figure 5a, the controlled manipulation of rotation speed, ball-to-powder ratio, and milling atmosphere enables the tailored modification of LiBH_4_ structure and properties [75]. The process involves repeated cycles of particle deformation, fracture, and cold welding under high-energy impacts (Figure 5b), leading to the creation of high-energy surfaces and defect-rich regions that enhance hydrogen storage kinetics [76]. Agresti et al. [77] demonstrated the remarkable potential of mechanochemical processing through their investigation of LiBH_4_ synthesis directly from LiH and B precursors. Their innovative approach utilized high-energy ball milling under a hydrogen atmosphere, revealing a direct correlation between milling duration and hydrogen release capacity. The process effectiveness was further enhanced through careful optimization of hydrogen pressure within the milling vial and the strategic substitution of WC balls for conventional stainless steel media. This methodology resulted in the successful synthesis of phase-pure LiBH_4_, which was confirmed through a detailed X-ray diffraction analysis of the separated crystalline product.

Complementary studies by Çakanyıldırım et al. [78] advanced the understanding of mechanochemical processing through the systematic investigation of different milling media and environments. Utilizing an SPX-type mill with various container materials (WC, stainless steel, and zirconia), they examined the impact of Li and B mixing ratios under a controlled argon atmosphere. An initial XRD analysis confirmed the formation of LiB intermediates, leading to the development of a specialized hydrogenation system operating at 60 bar pressure to facilitate hydrogen incorporation into the LiB structure. Optimization of the B/Li molar ratio to 0.214 resulted in successful LiBH_4_ formation, which was verified through comprehensive FTIR spectroscopic analysis. The hydrogen-rich nature of the resulting structure was further confirmed through simultaneous thermal analysis combining DSC and TGA techniques, demonstrating enhanced mass loss characteristics.

The incorporation of support materials during ball milling has proven crucial for controlling nanostructure evolution and preventing agglomeration. Zhang et al. [65] made significant advances in this area through the strategic use of few-layer graphene (FL-Grs) supports to modify LiBH_4_ morphology. Their work demonstrated a remarkable transformation from nanorods to nanoparticles in the presence of FL-Grs, with the resulting particles exhibiting controlled dimensions of 20–50 nm. This architectural modification enabled impressive performance metrics: hydrogen desorption initiated at 230 °C with absorption possible at 190 °C, accompanied by excellent cycling stability. Detailed mechanistic studies identified the growth and cyclic separation of B and LiH particles as key factors influencing the gradual evolution of desorption and absorption kinetics.

The optimization of ball milling processes for LiBH_4_ nanostructuring continues to face several technical challenges. Extended milling can lead to particle reagglomeration, necessitating careful process optimization. Wear from milling media and potential atmospheric exposure requires stringent control measures. The translation of laboratory-scale processes to industrial production presents significant engineering challenges, while maintaining nanostructured features during hydrogen cycling requires careful material design and processing control. Ongoing research is focused on developing advanced characterization techniques to monitor structural evolution during milling and optimizing processing parameters for enhanced performance and scalability.

### 4.2. Melt Infiltration Design

Melt infiltration has emerged as a sophisticated approach for engineering nanoconfined LiBH_4_ systems, offering precise control over material distribution and interface formation [79,80]. This technique exploits the melting behavior of LiBH_4_ to achieve intimate contact with host materials, enabling the creation of well-defined nanostructures with enhanced hydrogen storage properties. The strategic selection of porous support materials and careful control of processing conditions allow for the development of architectures that fundamentally alter the thermodynamic and kinetic landscape of hydrogen storage reactions.

Carbon-based materials have demonstrated exceptional promise as hosts for melt-infiltrated LiBH_4_ due to their thermal stability, tunable porosity, and surface chemistry. Guo et al. [66] achieved significant breakthroughs using carbon nanocages (CNCs) as host structures. Their LiBH_4_@CNCs composites exhibited dramatically reduced hydrogen release temperatures, initiating desorption at 200 °C with peak activity around 320 °C—representing a remarkable 180 °C reduction compared to bulk LiBH_4_. The system achieved a substantial hydrogen desorption capacity of 7.5 wt.%, maintaining 78% of initial capacity after five cycles at 400 °C. The enhanced performance stemmed from a complex interplay between nanoconfinement effects and chemical interactions. During infiltration, LiBH_4_ reacted with oxygen-containing functional groups on the CNC surface, forming LiBO_2_ and subsequently Li_3_BO_3_. These oxide species participated in additional reactions with LiBH_4_, generating supplementary hydrogen release pathways and enhancing the system’s reversible storage capacity. Surrey et al. [28] advanced the field through innovative template design, employing salt-templating methods to create precisely engineered carbon scaffolds. This sustainable approach to producing micro- and mesoporous carbon architectures enabled high LiBH_4_ loading capacities up to 40 wt.%. The nanoconfined system demonstrated hydrogen desorption initiation at 200 °C, with primary release occurring at 310 °C. While partial rehydrogenation was achieved under moderate conditions (100 bar, 300 °C), NMR investigations revealed the presence of amorphous and partially oxidized boron species in dehydrogenated samples, highlighting the complex relationship between host structure and reversibility. Liu et al. [67] conducted a systematic investigation into the fundamental effects of pore size on LiBH_4_ behavior within porous hard carbon templates. Their work revealed profound size-dependent effects on structural phase transitions and melting thermodynamics. In confined systems with pore dimensions below approximately 4 nm, conventional phase transitions and melting phenomena were progressively suppressed and ultimately eliminated. The desorption characteristics showed systematic improvement with decreasing pore size, accompanied by a notable reduction in B_2_H_6_ evolution. This suppression of borane formation indicated that spatial confinement could effectively block undesired reaction pathways, potentially improving the reversible storage capacity of the composites. Zhou et al. [68] demonstrated the effectiveness of activated carbon (AC) as a host material through careful melt infiltration studies. Their LiBH_4_/AC composites initiated hydrogen release at the remarkably low temperature of 190 °C—a 160 °C improvement over bulk LiBH_4_. The system achieved an impressive total hydrogen release of 13.6 wt.% upon heating to 400 °C. Under moderate rehydrogenation conditions (6 MPa H_2_, 350 °C), the composite maintained a reversible capacity of 6 wt.%, while unmodified LiBH_4_ showed negligible reversibility. The apparent activation energy for dehydrogenation decreased substantially from 156.0 kJ/mol to 121.1 kJ/mol, reflecting significant kinetic enhancement through nanoconfinement.

**Figure 6:**
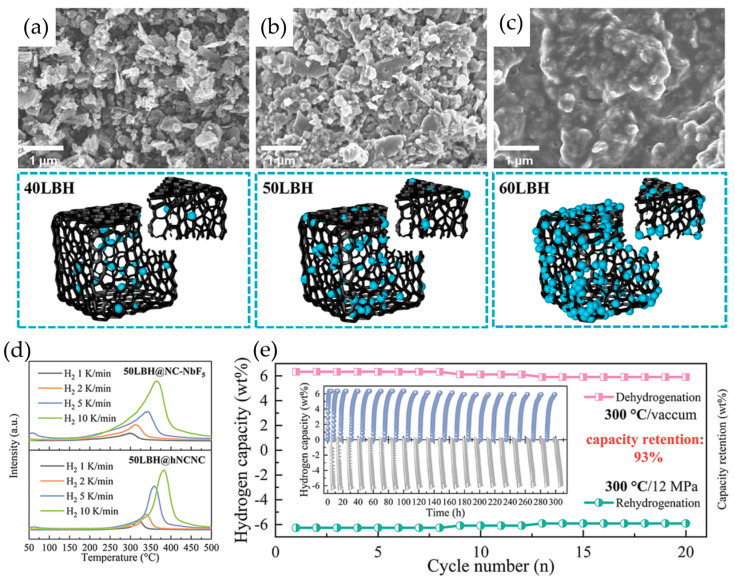
SEM of (**a**) 40LBH@NC-NbF_5_, (**b**) 50LBH@NC-NbF_5_, and (**c**) 60LBH@NC-NbF_5_ in the same magnification. (**d**) Theoretical and experimental dehydrogenation capacities of _x_LBH@hNCNC and xLBH@NC-NbF_5_ and their differences. (**e**) Cyclic performance with a regime of dehydrogenation at 300 °C in vacuum and rehydrogenation at 300 °C under 12 MPa H_2_ [69].

Further performance improvements have been realized through the strategic modification of carbon host materials. Gasnier et al. [81] investigated the effects of nitrogen doping in graphene-based hosts, infiltrating LiBH_4_ at various volume fractions (30%, 50%, and 70%). The N-doped system demonstrated a 10 °C reduction in release temperature accompanied by a 1 wt.% increase in hydrogen release at 325 °C. This enhancement proved more significant than geometric effects achieved by reducing pore dimensions from 10 nm to 5 nm, highlighting the importance of chemical modification in host design. Jia et al. [69] achieved remarkable results through the development of sophisticated N-doped carbon nanocages containing NbB_2_. Their systematic investigation of the xLBH@NC-NbF_5_ system revealed critical loading-dependent behavior. An SEM analysis (Figure 6a–c) demonstrated the preservation of the cage structure at 40 wt.% loading, with minor nanosheet fracturing occurring at 50 wt.%. At 60 wt.% loading, excess LiBH_4_ (26.8 wt.%) led to intersheet agglomeration. The optimized 50LBH@NC-NbF_5_ system achieved exceptionally low dehydrogenation temperatures and reduced activation energies (82.51 ± 6.46 kJ/mol), releasing 7.55 wt.% H_2_ within 39 min at 320 °C. Most impressively, the system retained 93% capacity through 20 cycles at 300 °C, demonstrating exceptional stability and reversibility.

The remarkable success of melt infiltration techniques in enhancing LiBH_4_ performance stems from multiple synergistic effects. The confined geometry restricts particle growth and agglomeration during cycling, while the intimate contact between LiBH_4_ and host materials facilitates heat and mass transfer. Additionally, the modified surface chemistry of host materials can influence local bonding environments and reaction pathways, potentially suppressing the formation of stable intermediates. These combined effects enable significant improvements in both the thermodynamic and kinetic aspects of hydrogen storage, though challenges remain in optimizing loading levels, preventing structural degradation during cycling, and maintaining long-term stability of the confined systems.

### 4.3. Solution-Based Synthesis

While melt infiltration has demonstrated considerable success in creating nanostructured LiBH_4_ systems, the high processing temperatures required can potentially compromise host material integrity and limit architectural control [82,83]. Solution-based impregnation methods have emerged as a sophisticated alternative, offering precise control over material distribution and morphology under mild conditions. This approach enables both the uniform incorporation of LiBH_4_ within porous frameworks and the synthesis of unsupported nanostructures through carefully controlled solution chemistry.

The versatility of wet chemical methods in modifying carbon-based host materials has been extensively demonstrated through systematic studies. Liu et al. [67] conducted pioneering work examining the confinement effects of porous hard carbon templates with varying pore architectures. Their research revealed that the phase behavior of confined LiBH_4_ underwent dramatic modifications compared to the bulk material. The characteristic phase transitions and melting phenomena of LiBH_4_ exhibited systematic shifts toward lower temperatures as pore dimensions decreased, eventually disappearing completely in pores smaller than approximately 4 nm. This geometric control extended beyond simple phase behavior, affecting the fundamental desorption characteristics. Notably, the evolution of B_2_H_6_, a problematic byproduct in LiBH_4_ decomposition, showed progressive suppression with decreasing pore size, indicating that spatial confinement could effectively modify reaction pathways and potentially enhance system reversibility. Eymery et al. [70] advanced the field through precise control of mesoporous carbon template architecture, developing host materials with optimized 4 nm pore dimensions and substantial pore volume (1.1 cm^3^ g⁻^1^). This careful structural design led to remarkable improvements in hydrogen desorption kinetics, with confined systems achieving 3.4 wt.% release within 90 min at 300 °C—conditions under which unmodified LiBH_4_ showed negligible decomposition. Their work with LiBH_4_/carbon composites at a 33:67 ratio revealed a simplified dehydrogenation pathway occurring above the LiBH_4_ melting point (280 °C), notably avoiding the formation of problematic intermediates such as dodecaborane. This streamlined decomposition route offered valuable insights into the relationship between confinement architecture and reaction mechanisms.

The application of wet chemical methods to oxide-based hosts has opened new avenues for performance enhancement. Liu et al. [71] developed an innovative approach using solvothermally synthesized porous TiO_2_ microtubes as host structures. The successful embedding of LiBH_4_ nanoparticles within the TiO_2_ framework through chemical impregnation demonstrated the synergistic benefits of nanoscale confinement and oxide catalysis. The LiBH_4_@2TiO_2_ system, optimized at a 1:2 mass ratio, achieved hydrogen release initiation at 180 °C, with the apparent activation energy reduced significantly from 146 kJ mol⁻^1^ to 121.9 kJ mol⁻^1^. Xu et al. [72] further expanded the potential of oxide hosts through their investigation of LiBH_4_ confined within porous NiMnO_3_ microspheres. The wet impregnation approach enabled the uniform distribution of LiBH_4_ within the NiMnO_3_ pore network, resulting in substantial improvements in dehydrogenation behavior. The composite system demonstrated impressive low-temperature performance, initiating hydrogen release at 150 °C and achieving a release capacity of 2.8 wt.% within 1 hour at 300 °C.

**Figure 7:**
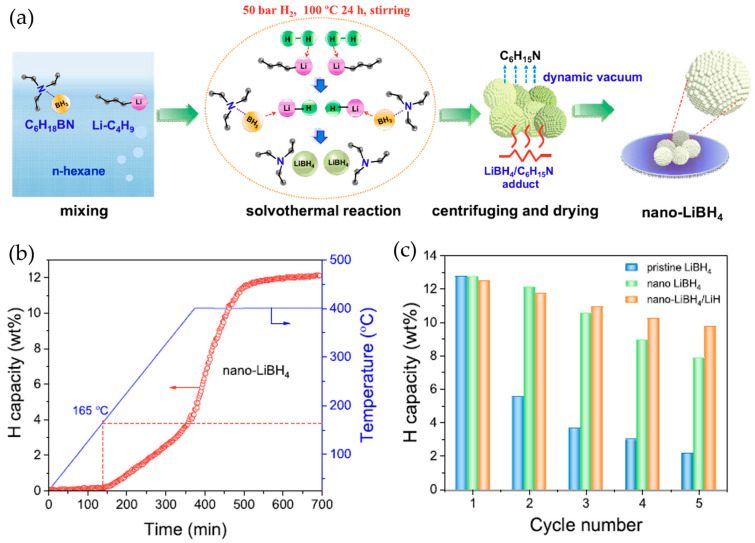
(**a**) Schematic of the preparation. (**b**) Non-isothermal hydrogenation curves of nano-LiBH_4_. (**c**) Comparison of dehydrogenation capacity of pristine LiBH_4_ (500 °C), nano-LiBH_4_ (400 °C), and nano-LiBH_4_/LiH (400 °C) with cycling [73].

Perhaps the most significant advancement in wet chemical processing has been the development of unsupported LiBH_4_ nanostructures. Zhang et al. [73] pioneered an innovative synthesis approach utilizing n-BuLi and Et_3_N·BH_3_ precursors in the n-hexane medium. Their sophisticated protocol, involving controlled sonication and thermal treatment under 50 bar H_2_ pressure at 100 °C, yielded hierarchically porous LiBH_4_ nanostructures composed of primary particles in the 50–60 nm range. This architectural design enabled remarkable performance improvements, with hydrogen desorption initiating at just 165 °C and achieving approximately 12 wt.% H_2_ release upon heating to 400 °C (Figure 7b). The engineered nanostructure demonstrated superior cycling stability compared to conventional LiBH_4_, exhibiting significantly reduced capacity decay over multiple cycles (Figure 7c).

The enhanced performance characteristics achieved through wet chemical processing stem from several key advantages of the approach. The low-temperature conditions preserve the structural integrity of both host materials and the resulting nanostructures. Solution-phase processing enables precise control over particle size and morphology while also facilitating uniform distribution within host frameworks. The technique allows for the incorporation of surface modifiers and stabilizers that can influence interfacial properties and stability. However, the method requires careful control of processing parameters to ensure complete solvent removal and prevent oxidation or degradation during synthesis. The relationship among processing conditions, resulting architecture, and hydrogen storage performance continues to be an active area of investigation, with particular emphasis on optimizing structure stability and cycling behavior.

## 5. Reactive Composite Systems

Reactive hydride composites (RHCs) represent an advanced strategy for fundamentally altering the thermodynamic and kinetic landscape of LiBH_4_-based hydrogen storage systems. This approach strategically combines LiBH_4_ with secondary hydrides to establish alternative reaction pathways through the formation of thermodynamically favorable compounds during dehydrogenation [37,84,85]. The underlying principle relies on the creation of more stable dehydrogenated products (such as MgB_2_, CaB_6_, or AlB_2_) compared to elemental boron, effectively reducing the overall reaction enthalpy and lowering hydrogen release temperatures. These newly formed phases not only modify the system’s thermodynamics but also create dynamic interfaces that enhance hydrogen diffusion and mass transfer. The presence of multiple hydride phases generates additional active sites for hydrogen interactions, while the structural evolution during cycling introduces beneficial defects and high-energy interfaces, collectively contributing to improved kinetics and reversibility while maintaining high storage capacity.

### 5.1. LiBH_4_-MgH_2_ Composites

The combination of LiBH_4_ with MgH_2_ has emerged as a particularly promising RHC system, offering significant advantages through cooperative dehydrogenation processes and the formation of stable reaction products. Systematic investigations have revealed complex reaction mechanisms that can be strategically manipulated to enhance system performance. Bösenberg et al. [86] conducted pioneering studies on the decomposition pathways of LiBH_4_-MgH_2_ composites under varying pressure and temperature conditions, revealing distinct reaction regimes: at 450 °C and 3 bar, MgH_2_ and LiBH_4_ underwent separate decomposition, while at 400 °C and 5 bar, simultaneous hydrogen release from LiBH_4_ occurred alongside MgB_2_ formation. In situ X-ray diffraction and infrared spectroscopy demonstrated the presence of both crystalline and amorphous phases during reaction progression, notably without detecting intermediate metallic Mg phases during hydrogen evolution.

**Figure 8:**
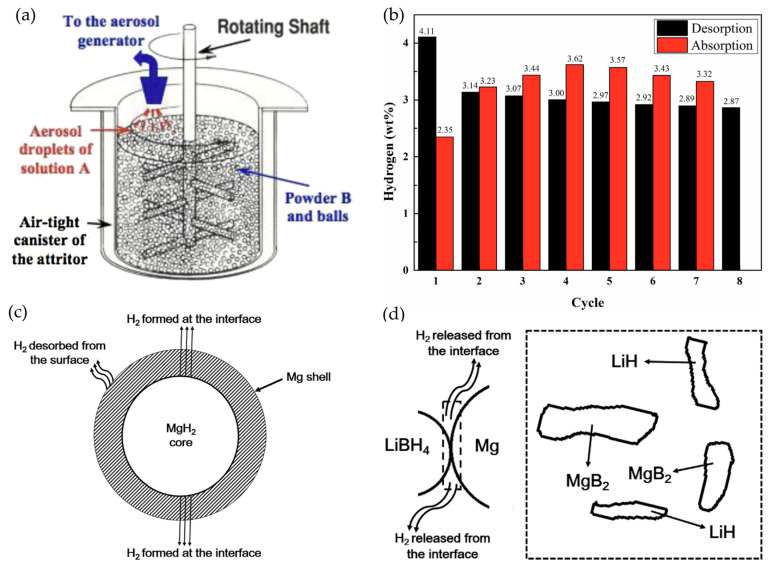
(**a**) Schematic of the device for the ball milling with aerosol spraying (BMAS) process in making nano-particles with reactants LiBH_4_ and MgH_2_ mixing at the nanometer scale [88]. (**b**) Hydrogen amount of desorption and absorption as a function of dehydrogenation and re-hydrogenation cycles [90]. Schematic of the dehydrogenation via the LiBH_4_-Li_2_B_12_H_12_ pathway: (**c**) the (Li_2_B_12_H_12_ + 10 LiH) products form a continuous shell outside the LiBH_4_ shrinking core, leading to a reaction rate controlled by H_2_ gas desorption at the surface of the (Li_2_B_12_H_12_ + 10 LiH) product layer, and (**d**) takes place at the Li_2_B_12_H_12_/MgH_2_ interface, leading to the nucleation and growth of MgB_2_ + LiH products (shown inside the dashed box) accompanied by H_2_ release [92].

The development of advanced processing techniques has led to significant breakthroughs in system performance. Crosby et al. [87] demonstrated remarkable improvements through low-temperature graphite addition during high-energy ball milling of 2LiBH_4_ + MgH_2_ mixtures, achieving a fivefold increase in hydrogen release at 265 °C compared to conventionally milled systems. Both hydride components contributed to solid-state hydrogen release, with rehydrogenation kinetics primarily controlled by Mg reformation. These enhancements were attributed to the formation of nanocrystalline and amorphous phases, increased defect concentrations, and potential catalytic effects of the various system components. Building on these advances, Ding et al. developed the innovative Ball Milling Aerosol Spraying (BMAS) technique, illustrated in Figure 8a, which integrates aerosol generation with ball milling to enable precise control over particle formation in the 0.02–0.30 mm range [88]. This process facilitates the room-temperature formation of Li_2_B_12_H_12_ through aerosolized LiBH_4_/THF solution decomposition, followed by the reaction with in situ formed MgH_2_ to produce MgB_2_ and LiH [89].

The BMAS-processed LiBH_4_ + MgH_2_ nanocomposite demonstrated exceptional performance through a complex network of parallel hydrogen release mechanisms, involving the decomposition of nano-LiBH_4_, nano-Mg(BH_4_)_2_, and nano-MgH_2_ [90]. This sophisticated reaction architecture enabled the solid-state hydrogen release of 4.11 wt.% at ≤265 °C, maintaining 2.87 wt.% capacity after eight cycles [91]. The detailed kinetic analysis, illustrated in Figure 8c,d, revealed a fascinating evolution in the rate-limiting step from nucleation/growth to moving phase boundary control and eventually to diffusion control with increased cycling [92]. The formation of a continuous Mg shell around the shrinking MgH_2_ core during dehydrogenation emerged as a critical architectural feature, controlling hydrogen diffusion rates and directly impacting system performance through the modulation of the interfacial area between MgH_2_ and LiBH_4_.

The remarkable success of the LiBH_4_-MgH_2_ system stems from the synergistic combination of thermodynamically favorable MgB_2_ formation during dehydrogenation, the creation of active interfaces that facilitate mass transport, and the stabilization of reaction intermediates through controlled phase evolution. These fundamental advances in understanding and controlling the complex interplay between processing, structure, and performance have established a robust foundation for the continued development of practical hydrogen storage systems based on reactive hydride composites.

### 5.2. Complex Hydride Combinations

The development of alternative reactive hydride composites beyond MgH_2_-based systems has revealed promising pathways for enhancing the hydrogen storage performance of LiBH_4_. Complex hydrides such as NaAlH_4_ and LiAlH_4_ offer unique advantages through their relatively low dehydrogenation temperatures, favorable thermodynamics, and ability to form stable intermediates that promote reversible hydrogen storage. These systems demonstrate distinct reaction mechanisms and synergistic effects that contribute to improved overall performance characteristics.

NaAlH_4_-based systems have demonstrated particularly promising results through carefully designed compositional control. Yap et al. [93] conducted comprehensive investigations of Na_3_AlH_6_-LiBH_4_ composites with varying stoichiometric ratios, revealing unexpected reaction pathways that surpassed conventional double decomposition mechanisms. Their systematic study of 1:3 and 1:4 molar ratio composites demonstrated the formation of Li_3_AlH_6_ and NaBH_4_ phases, with the 1:4 ratio exhibiting superior hydrogenation performance. The system’s dehydrogenation proceeds through a sophisticated three-stage mechanism: initial hydrogen release begins at 180 °C with Li_3_AlH_6_ decomposition to LiH and Al; subsequently, NaBH_4_ decomposition occurs around 380 °C, coupled with an Al reaction to form AlB_2_ at a temperature approximately 100 °C lower than isolated NaBH_4_; the final stage involves catalytic decomposition of residual NaBH_4_ with AlB_2_ at 430 °C. Under moderate conditions (320 °C, 33 atm H_2_), the composite demonstrates rapid hydrogen absorption, achieving 3.0 wt.% within 60 min, with capacity increasing to 6.0 wt.% when heated to 420 °C.

The incorporation of advanced nanostructuring strategies has further enhanced the performance of NaAlH_4_-based composites. Javadian et al. [94] achieved significant improvements by confining 2LiBH_4_-NaAlH_4_ within a mesoporous carbon aerogel framework (pore size: 30 nm, BET surface area: 689 m^2^/g, total pore volume: 1.21 mL/g). This architectural modification reduced the hydrogen release temperature by 132 °C compared to bulk material, enabling hydrogen evolution below 100 °C. The confined system undergoes a complex reaction sequence involving LiAlH_4_ and NaBH_4_ formation, ultimately producing LiH, LiAl, AlB_2_, and Al as dehydrogenation products. Rehydrogenation studies at 400 °C and 126 bar demonstrated the recovery of amorphous NaBH_4_ alongside unreacted species, identifying NaBH_4_ as a crucial component for reversible hydrogen storage. The nanoconfined system maintained 83% of initial hydrogen content after three cycles, substantially outperforming the bulk material’s 47% retention.

LiAlH_4_-based systems offer complementary advantages through their structural similarity to LiBH_4_ and distinct reaction characteristics. Li et al. [95] demonstrated the effective destabilization of LiBH_4_ using both LiAlH_4_ and Li_3_AlH_6_, with 2LiBH_4_-Li_3_AlH_6_ and 2LiBH_4_-LiAlH_4_ systems achieving dehydrogenation at 416 °C and 435 °C respectively—significantly below pure LiBH_4_’s 469 °C. The composites released substantial hydrogen quantities, with 2LiBH_4_-Li_3_AlH_6_ achieving a 9.1 wt.% H_2_ release within 150 min, demonstrating superior kinetic performance. Meethom et al. [96] further enhanced reaction kinetics through an innovative approach combining dehydrogenated composite quenching and re-milling. This process promoted reactions between Al and both LiBH_4_ and LiH, yielding AlB_2_ and LiAl phases that significantly improved system performance—tripling kinetic rates and reducing the initial temperature by 120 °C while maintaining reversibility.

The incorporation of catalytic additives has proven effective in further optimizing LiAlH_4_-LiBH_4_ systems. Mao et al. [97] demonstrated remarkable improvements through TiF_3_ addition, achieving reductions in initial dehydrogenation temperatures of 64 °C and 150 °C for the first two stages respectively, compared to the undoped system. The catalyst facilitated Al-B reactions to form AlB_2_, providing additional active sites and reducing the decomposition enthalpy from 74 kJ/mol H_2_ to 60.4 kJ/mol H_2_. Under optimized conditions (600 °C, 4 MPa H_2_), the system achieved hydrogen absorption capacities of 3.76 wt.% within 1 h and 4.78 wt.% after 14 h, demonstrating practical rehydrogenation capabilities.

The diverse performance characteristics of these alternative reactive hydride composites highlight the potential for tailored system design through the careful selection of constituent hydrides and processing conditions. The formation of intermediate phases, coupled with strategic architectural control and catalyst incorporation, enables the development of systems with improved thermodynamics, enhanced kinetics, and maintained reversibility. These advances in complex hydride combinations continue to expand the possibilities for practical hydrogen storage applications based on LiBH_4_ systems.

## 6. Research Gaps and Conclusion Remarks

Although significant advances have been achieved in LiBH4-based systems, several critical research gaps persist that hinder their practical implementation. At the fundamental level, the interface dynamics during hydrogen cycling remain incompletely understood, particularly regarding the stability and evolution of catalytic interfaces over extended cycling periods. The complex interactions between catalysts and the host material, especially under dynamic cycling conditions, require deeper investigation. While the formation of Li_2_B_12_H_12_ and other intermediates has been widely observed, the precise mechanisms controlling their formation, stability, and decomposition pathways are not fully understood, making it challenging to develop effective strategies for enhancing system reversibility. The role of local chemical environments and defect structures in influencing intermediate phase formation needs systematic study.

From a materials engineering perspective, significant challenges remain in optimizing the synergistic effects between different modification strategies. The interaction between catalytic additives and nanoconfinement effects, for instance, requires further investigation to maximize their combined benefits. Most reported advances focus on laboratory-scale demonstrations, leaving significant knowledge gaps in scaling up these improvements to practically viable systems. Critical questions remain regarding heat management in larger systems, pressure distribution in scaled-up configurations, and material containment strategies for practical applications. Current characterization methods primarily rely on ex-situ analysis, limiting our understanding of real-time material evolution during hydrogen cycling. The development of advanced in operando characterization techniques is essential for understanding the dynamic processes occurring during hydrogen storage and release.

Additionally, the long-term stability and degradation mechanisms of modified LiBH_4_ systems under realistic operating conditions remain inadequately explored. The impact of thermal cycling, mechanical stress, and environmental factors on system performance needs systematic investigation. The development of standardized testing protocols and performance metrics that accurately reflect real-world application requirements represents another critical research gap.

The systematic investigation of LiBH_4_-based hydrogen storage materials has demonstrated that rational material design and optimization can substantially enhance system performance through three distinct modification strategies: catalytic incorporation, nanostructure engineering, and reactive composite development.

Catalytic modification strategies have evolved from simple dopant addition to sophisticated interface and electronic structure engineering. Early studies focusing on transition metal catalysts have expanded to include advanced carbon architectures and metal oxide systems, revealing the critical role of surface chemistry and interface dynamics. The most significant advances have emerged from understanding the mechanistic aspects of catalytic enhancement, particularly how different catalyst types influence specific reaction steps. Advanced carbon frameworks demonstrate exceptional performance through their tunable surface properties and high active site density, while metal oxide systems excel in stabilizing reaction intermediates and modifying local electronic environments. The development of hybrid catalytic systems has further revealed synergistic effects that can simultaneously address multiple performance limitations.

Nanostructure engineering has progressed from basic size reduction to precise architectural control, fundamentally altering the thermodynamic and kinetic landscape of LiBH_4_ systems. The field has witnessed a transition from conventional ball milling to sophisticated templating and infiltration techniques, enabling unprecedented control over material architecture across multiple length scales. Critical insights have emerged regarding the relationship between structural features and system performance, particularly how confined geometries and engineered interfaces influence hydrogen storage behavior. The development of hierarchical architectures combining multiple structural features has proven especially effective in optimizing overall system performance, though maintaining structural integrity during cycling remains challenging.

Reactive composite development has evolved from simple mixture studies to carefully designed multi-component systems that achieve fundamental modifications of reaction thermodynamics. The success of LiBH_4_-MgH_2_ systems has demonstrated how strategic phase formation can enhance reversibility while maintaining high capacity, while investigations of complex hydride combinations have revealed complementary approaches to thermodynamic destabilization. The field has progressed toward understanding the complex interplay among phase evolution, interface formation, and reaction kinetics, enabling more rational design of composite systems.

The advancement of these modification strategies has been significantly enhanced by the development of advanced characterization techniques and improved theoretical understanding. The integration of multiple approaches has revealed promising synergistic effects, while the systematic investigation of structure–property relationships has enabled more rational material design. As summarized in Table 5, each strategy has undergone significant evolution, achieved specific breakthroughs, and faced distinct challenges. The accumulated knowledge and understanding provide a strong foundation for the future development of practical LiBH4-based hydrogen storage systems.

Rather than operating independently, these three strategies increasingly demonstrate complementary effects when appropriately combined. The future advancement of LiBH_4_-based systems will likely depend on the strategic integration of multiple modification approaches while addressing their respective limitations. The progress achieved thus far, while substantial, also highlights the importance of continued fundamental research alongside practical engineering solutions.

## 7. Future Perspectives

The transition toward practical applications of LiBH_4_-based hydrogen storage systems faces multiple fundamental challenges requiring systematic investigation and innovative solutions. From an economic perspective, the scaled production of engineered materials, particularly nanostructured composites and advanced catalysts, must achieve cost-effectiveness while maintaining critical performance characteristics. The optimization of synthesis routes and material selection needs to balance enhanced storage properties with manufacturing feasibility and material costs.

Technical barriers present significant challenges for practical implementation. Current operating temperatures exceeding 300 °C remain substantially higher than the target range below 100 °C required for most applications. Kinetic limitations during both hydrogen absorption and desorption processes impact system response times and cycling efficiency. The development of efficient heat management strategies during operation is crucial, as thermal effects significantly influence both performance and system stability. Additionally, maintaining long-term stability under practical operating conditions requires careful consideration of material degradation mechanisms and their mitigation.

The scale-up of laboratory processes to industrial production presents complex engineering challenges. Maintaining critical material features during large-scale synthesis while ensuring reproducibility and quality control requires sophisticated process optimization. The integration of storage systems with fuel cell technologies demands careful consideration of interface management and system compatibility. The development of standardized testing protocols for practical evaluation becomes increasingly important for reliable performance assessment. 

Looking forward, several promising research directions emerge for addressing these challenges. Advanced material design strategies focus on developing novel multi-functional catalysts and engineered interfaces that can enhance kinetics while maintaining stability. The implementation of in situ and operando characterization techniques provides deeper insights into material evolution during cycling, enabling more effective system optimization. Computational modeling approaches offer powerful tools for material screening and mechanism investigation under practical conditions.

System integration represents a critical area for future development. The optimization of heat exchange systems and pressure management strategies must address both performance and safety considerations. Modular design approaches offer flexibility for various applications while maintaining system efficiency. The successful development of practical LiBH_4_-based storage systems ultimately requires coordinated efforts across multiple research domains, combining fundamental understanding with engineering solutions while maintaining economic viability. 

## Figures and Tables

**Table 1:** US DOE technical targets for onboard hydrogen storage systems.

Storage System Targets	Gravimetric Density (kg H_2_/kg System)	Volumetric Density (kg H_2_/L System)	Cost $/kWh ($/kg H_2_)
2020	1.5	1	$10
	−0.045	−0.03	($333)
Ultimate	2.2	1.7	$8
	−0.065	−0.05	($266)

**Table 2:** Properties of LiBH_4_ as hydrogen storage material.

Advantages	Disadvantages
High theoretical capacity (18.5 wt.%)	High dehydrogenation temperature (>400 °C)
Abundant constituent elements	Slow kinetics for H_2_ release/uptake
Good theoretical volumetric density	Formation of stable intermediates (Li_2_B_12_H_12_)
Relatively low material cost	Poor reversibility
Good stability at room temperature	Complex multi-step reaction pathway
High volumetric density	Severe rehydrogenation conditions (>600 °C, >150 bar)

**Table 5:** Comprehensive analysis of LiBH_4_ modification strategies from fundamental mechanisms to system performance.

Strategy	Scientific Foundation	Design Evolution	Key Breakthroughs	System Impact	Current Limitations
Catalytic modification	Surface chemistry and electronic modification	Single dopant to multi-functional catalysts	Hybrid catalyst systems; interface engineering	Temperature reduction: 100–150 °C; enhanced kinetics	Long-term stability; Cost-effectiveness
Nanostructure engineering	Size effects and interface physics	Mechanical processing to templated synthesis	Hierarchical architectures; confined geometries	Onset temperature: ~200 °C; modified thermodynamics	Structure preservation; scale-up challenges
Reactive composites	Thermodynamic destabilization	Binary mixtures to multi-component systems	Strategic phase formation; synergistic effects	High capacity (8–11 wt.%); enhanced reversibility	Complex phase evolution; heat management

## Data Availability

Not applicable.
